# The predictive role of tendency toward mobile learning and emotional intelligence in Internet addiction in healthcare professional students

**DOI:** 10.30476/JAMP.2022.91971.1465

**Published:** 2022-04

**Authors:** NAYEREH BAGHCHEGHI, HAMID REZA KOOHESTANI

**Affiliations:** 1 Social Determinants of Health Research Center, Saveh University of Medical Sciences, Saveh, Iran

**Keywords:** Internet addiction disorder, Emotional intelligence, Healthcare, Students

## Abstract

**Introduction:**

Internet addiction is a psychological disorder that can lead to serious damages to university students as a group at risk. This study aimed to determine the predictive role of tendency toward mobile learning (purposeful use of mobile technologies for educational purposes) and emotional intelligence in Internet addiction in healthcare professional students.

**Methods:**

A cross-sectional descriptive study was carried out on 403 healthcare professional students at Saveh University of Medical Sciences-Iran in 2021 using convenience sampling method. For data gathering, three questionnaires were used: willingness to use mobile learning, Young’s Internet Addiction Test, and the Emotional Intelligence Appraisal. Data analyses were done using Pearson Correlation Coefficient and Hierarchical Regression in SPSS 16 (Inc SPSS USA, IL, Chicago).

**Results:**

The mean age of the participants was 21.09±1.47 years; 125 subjects (31.01%) were boys and 278 (68.99%) girls. As the findings showed, 16.87% of the students had Internet addiction and 33% were on the edge of developing Internet addiction. Internet addiction was significantly negatively correlated with willingness to m-learning (r=-0.45, P=0.001) and emotional intelligence (r=-0.32, P=0.01). In addition, regression analysis results showed that the variables willingness to use learning and emotional intelligence explained 23% of the Internet addiction variance (P<0.001).

**Conclusion:**

In the present study, a considerable number of the healthcare professional students had excessive and unnecessary use of the Internet. Emotional intelligence and willingness to use mobile learning had an inverse relationship with Internet addiction. There is a need to screen Internet-addicted students using proper screening tools and take primary preventive measures in this regard. In addition, proper measures are needed to be taken to improve emotional intelligence and mobile learning skills and control Internet addiction to some extent.

## Introduction

Internet addiction, also known as problematic Internet use, is an excessive and inconsistent pattern of Internet use that leads to significant impairment or distress ( 1 ). Using the Internet in the right way is surely beneficial; however, problems arise when it is used in an uncontrolled manner or when it becomes an addiction ( 2 ). In comparison to adults, university students and adolescents are at a higher risk of developing Internet addiction ( 3 , 4 ). Individuals in this age group are naturally interested in the Internet and this can lead to Internet addiction. Normally, adolescents have more free time, easier access to the Internet, and minimum parental control ( 5 ). Addictive behavior can lead to a major problem during lock-down periods ( 4 ). It is highly prevalent among the university students, so that a study in Iran reported over9.8% of healthcare professional students were Internet addicts ( 1 ). In the study of Ghahremani et al., 6.7% of medical sciences students had dependence on the Internet ( 6 ). The issue of Internet addiction can be considered a social crisis and has attracted the attention of experts in all fields. This phenomenon is a biological, psychological, social, economic and cultural problem, and the issue of Internet addiction cannot be viewed only from one perspective; it is actually affected by many factors and causes many negative consequences ( 7 ). Students who are addicted to the Internet have an excessive and problematic use of the Internet, which can lead to disorders in their mental health and academic performance ( 8 ). The results of a study showed that Internet addiction reduces the quality of medical sciences students' sleep and increases their anxiety ( 9 ).

Mobile devices such as smartphones are featured with a wide range of functionalities that converge different technologies. These devices have created an unprecedented and exciting opportunity in teaching/learning field ( 10 , 11 ). After expansion of digital culture and popularization of modern forms of communications, we are witnessing the emergence of new terms and concepts. One of them is mobile learning (m-learning) ( 12 , 13 ), which means using mobile electronic devices for educational objectives ( 14 ). The key aspect of m-learning, according to the literature, is learning without place and time limitation ( 10 , 13 ). The method of using the media has a role to play in Internet addiction ( 15 ). Thus, the relationship between willingness to mobile learning (i.e., a proper way of using mobile technology for educational purposes) and Internet addiction was examined in this study.

The emotional intelligence (EI) includes one’s ability to see his/her own and others’ feelings and emotions to see differences between them and utilize such information to direct one’s thinking and actions. Having the right emotional intelligence allows a person to have proper emotional management and in interpersonal relationships to quickly and correctly process emotional data and take appropriate actions with emotions according to environmental conditions ( 16 ). According to the Interaction of Person-Affect-Cognition-Execution model, addictive Internet behaviors are affected by the interactions among predisposing factors (such as capabilities and traits including emotional intelligence). Such a behavior has a negative impact on one’s cognitive capabilities and affects reaction to situational triggers, which results in a decrease in executive functioning ( 3 ). People with low EI are at a higher risk of experiencing elevated interpersonal and psychological problems ( 17 ). Based on this model, the present study is an attempt to examine if emotional intelligence (EI) predicts addictive Internet use. Still, authors have reported contradictory results as to the type and strength of the relationship of these constructs. Further studies are needed to be conducted to confirm these claims ( 18 ). 

Given the Internet addiction in students can seriously damage their academic performance, it is necessary to examine the factors affecting this issue from different angles such as attitudes toward mobile learning and emotional intelligence, so that, if necessary, basic measures should be taken regarding prevention or treatment of Internet addiction. Thus, the present study is an attempt to examine the predictive role of willingness to use mobile learning and EI in Internet addiction of healthcare professional students. 

## Methods

### Study design

This is an analytical cross-sectional study on 403 health care professional students at Saveh University of Medical Sciences, Iran in 2021. 

### Participants

The study population consisted of all  undergraduate nursing, operation room, anesthesia, midwifery, prehospital emergency care, occupational health, public health, environmental health, and health information technology students in the second semester and higher by convenience sampling method. In this study, no sampling was performed and all eligible students in the research environment were invited to participate in the study by census method. These criteria were enrollment in the second semester or higher and willingness to take part in the study. The exclusion criterion was incomplete questionnaire. 

### Measurements

Data gathering was done using three questionnaires: willingness to mobile learning questionnaire, Young’s Internet Addiction Test, and the Emotional Intelligence Appraisal. The questionnaires were distributed as online forms. 

### Willingness to m-learning questionnaire

The willingness to m-learning questionnaire was designed by Baghcheghi et al. in 2020; it contains 45 questions with nine subscales (viz. technophilia, perceived attraction, perceived conflict, perceived ease, attitude, behavioral intention to use, self-management in learning, learning efficiency, and educational use). The scoring of each item is based on Likert five-point scale (completely agree, agree, disagree, completely disagree, and no idea). Total score ranges from 45 to 225 ( 10 ). The qualitative content validity of this questionnaire was checked using the comments and suggestions of 10 members of faculty board as experts in education and psychology. Also, In order to assesses the Content Validity Index (CVI), three criteria, including simplicity, specificity and clarity, were used for each question, on a 4-point scale. The item-level CVI (I-CVI) was evaluated by adding the number of experts who had scored the question as 3 or 4, divided by the total number of experts. An item is acceptable when the ICVI is higher than 79% and the item should be revised if the obtained ICVI is between 70 and 79. Items with ICVI < 70 should be removed. Based on the feedbacks, a few changes were made in 8 items of the questionnaire and no items were deleted. Reliability of the tool was assessed using internal consistency with Cronbach's alpha (0.88), and stability reliability was determined using test-retest (r=0.91) on 15 medical sciences students with a 2-week interval.

### The Emotional Intelligence Appraisal

The Emotional Intelligence Appraisal instrument was introduced in 2005 by Bradberry and Greaves with 28 items, which were categorized into four components. These components are social awareness, self-management, self-awareness, and relationship management. The questions are based on a six-point Likert-type scale as follows: “1=Never,” “2=Rarely,” “3=Sometimes,” “4=Usually,” “5=Almost Always,” and “6=Always”. The test has previously been translated into Persian and validated ( 19 , 20 ). In this study, content validation methods and Cronbach's alpha method (r=0.78) were used to determine the validity and reliability of the instrument, respectively.

### Internet Addiction Questionnaire

Young’s Internet Addiction Test (YIAT) was the third tool, which is a structural self-statement questionnaire to examine Internet addiction. The tool is widely used to measure Internet addiction. With 20 items, it is based on Likert five-point scale (rarely, …, always) with maximum score of 100. The higher the score, the higher the level of intensity of Internet addition. Based on the obtained score, the respondents are considered as normal Internet user [20-39], users with frequent [40-69] and significant problems [70-100] caused by Internet use ( 21 ). In this study, content validity and Cronbach's alpha (r=0.81) were used to determine the validity and reliability of the instrument. Given that online education is common during COVID-19 pandemic, students were told that using the Internet for educational purposes was not considered as Internet addiction.

### Data analysis

The data were analyzed in SPSS 16 software (SPSS Inc., Chicago, IL, USA) using Pearson’s Correlation Coefficient and Hierarchical Regression (confidence level = 95%, P-value=0.05).

### Ethical considerations

This study was conducted in accordance with the Declaration of Helsinki and approved by the Institutional Review Board and the Ethics Committee of Saveh University of Medical Sciences, Saveh, Iran (approval code: IR.SAVEHUMS.REC.1399.025). All participants were informed about the study objectives, their freedom to participate in or withdraw from the investigation. All participants gave electronic informed consent for participating and completing the questionnaires. 

## Results

Demographics characteristics of the participants are presented in Table 1. The mean score of Internet addiction, based on Young’s scale, was 66.07±23.94, which indicates a marginal level of Internet addiction (at risk). In Internet addition, 16.87% of the students had Internet addiction (score = 70-100) and 33% of them were at marginal range of Internet addiction (score = 40-69).

**Table 1:** Participants’ demographic characteristics (N = 403)

Variable	N	%
Gender		
Female	125	31.01%
Male	278	68.99%
Field of study		
Nursing	75	18.61
Midwifery	32	7.94
Operative room technology	53	13.15
Anesthesia	35	8.68
Occupational health	41	10.17
Public health	39	9.68
Environmental health	44	10.92
Health information technology	48	11.91
Prehospital emergency care	36	8.94
Age (years)		
Mean (SD)	21.09 (1.47)	

The mean score of EI of the participants was 68.53±12.36 (out of 100), which is indicative of “need help” category. The mean score of willingness to m-learning was 150.14±24.36, which indicates a moderate desire to m-learning. To examine the relationship between the variables, Pearson Correlation was used (Table 2). 

**Table 2:** Correlation matrix among willingness to m-learning, emotional intelligence, and Internet addiction

	Willingness to m-learning	Emotional intelligence
Willingness to m-learning	1	
Emotional intelligence	0.41**	1
Internet addiction	-0.45**	-0.32*

P=0.001 , P=0.02 , N=403

As listed in Table 1, Internet addiction was significantly negatively correlated with willingness to m-learning and emotional intelligence, showing that an increase in willingness to use m-learning and emotional intelligence leads to a decrease in Internet addiction. In other words, students with higher willingness to m-learning had a lower Internet addiction. In addition, students with better emotional intelligence had a lower internet addiction score. 

To examine the role of willingness to use m-learning and emotional intelligence to predict internet addiction, hierarchical multivariate regression was used (Table 3). The assumption of independent errors was checked using Durbin Watson test beforehand, which was obtained 1.72, supports the hypothesis (the range of 1.5 to 2.5 are relatively normal).

**Table 3:** Summary of regression analysis to predict Internet addiction based on willingness to use m-learning (ML) and emotional intelligence (EI)

No.	Predict	B	Beta	t	R	R2	Adjusted R2	F
1	Willingness to ML	-0.51	-0.45	-10.34**	0.45	0.20	0.2	106.9***
2	Willingness to ML	-0.39	-0.38	-8.15**	0.48	0.23	0.23	60.9***
	Emotional intelligence	-0.28	-0.16	-3.47*				
3	Willingness to ML	-0.35	-0.38	-7.99**	0.48	0.23	0.22	48.59***
	Emotional intelligence	0.31	-0.17	-1.37				
	ML×EI	0.02	0.09	0.50				

*** P < 0.001,** P < 0.01, *P < 0.05

As listed in Table 2, willingness to use m-learning was added to the equation as the predicting variable at the first step and 20% of Internet addiction variance was attributed to this variable. Then, emotional intelligence was added to the regression equation and 3% was added to the coefficient of determination, so that 23% of the variance of Internet addiction was attributed to two variables (P<0.001). 

Beta coefficient was significant in the first step (β= -0.45, t= -10.34, p< 0.01), indicating that willingness to use m-learning had a significant share in predicting Internet addiction. The Beta value of emotional intelligence in the second step was still significant (β= -0.16, t=-3.47, p<0.01), so that emotional intelligence had an independent role in predicting Internet addiction. The Beta value in the third step, i.e. the interaction of spiritual health and resilience (β= 0.02, t=0.9, p>0.05) on Internet addiction, was not significant (Table 3). 

## Discussion

The predictive role of willingness to m-learning and EI in Internet addition of healthcare professional students was examined. As the results showed, the majority of the participants were in marginal range (at risk) in Internet addition. Moreover, there was an inverse relationship between willingness to use m-learning and ET with Internet addiction. In other words, the higher score of willingness to use m-learning and EI can control Internet addiction. 

As to m-learning, the results showed that the majority of the participants had a positive perception about it, which is consistent with the results of other studies ( 12 , 22 ) . Another major finding of the study was the inverse relationship between m-learning and Internet addiction, which is not reported by other previous studies. By m-learning, we refer to a purposeful use of m-technologies for educational purposes, and willingness to use m-learning scale measures the level of willingness in students to use m-learning for educational purposes. Internet addiction refers to an unnecessary and unhealthy use of the Internet that can attenuate one’s ability to do daily tasks by wasting a great deal of time every day. However, willingness to use m-learning refers to one’s tendency for purposeful use of m-technology including smart phones for educational purposes. Therefore, one interpretation of the findings is that the students who use m-technology for education are at a lower risk of Internet addiction. Using m-mobile learning, learns can learn without time and place limitations, and during COVID-19 pandemic it can be a way to fill the educational gap. M-learning is an inevitable choice during COVID-19 pandemic ( 23 ). A study showed that willingness to m-learning had a direct relationship with educational achievement of healthcare professional students ( 24 ). There is no limit for Internet addiction and the time spent in social media and situation worsens with introduction of new mobile applications day by day. Therefore, it appears that the process of taming the technology is a continuous process. There is a need to educate the purposeful use of m-technology along with media literacy and help the students to identify purposeful techniques and methods of using the technology. Students need to learn selection skills and use them to achieve better outcomes. This can decrease media and Internet addiction. It is recommended, therefore, that educational programs should be held for students about purposeful use of m-learning towards educational purposes. 

As to the relationship between EI and Internet addition, the results of three studies have shown a negative relationship, which is consistent with our findings ( 25 - 27 ). In addition, results of another study showed a negative relationship between technology addiction and EI ( 28 ). Sechi et al. (2020) studied university students and showed that EL and self-esteem had a direct protective effect on Internet addiction behaviors ( 17 ). One of the main reasons for individuals’ tendency toward addiction is the major problems caused in emotional field. In fact, some individuals cannot control and manage their emotions or react properly to others’ emotions and they are at the higher risk of addiction. On the other hand, individuals with high EI tend to have a better perception of their own and others’ emotions and perform better through predicting the other’s expectations. They understand the unwanted peer pressure and are better in controlling their emotions, so that they are more resistive to addiction ( 29 ). Considering the fact that EI is an acquirable and improvable skill and according to the results obtained in this study, we suggest that the students with low EI must be screened and trained about EI to prevent Internet addition among them.

The present study has some strengths and limitations. In summary, the results are interesting from several aspects. First of all, the results indicated a relatively high prevalence of Internet addiction among healthcare professional students, which requires paying more attention to one’s behaviors and adopting solutions to prevent such behaviors. Students at risk need to be screened properly and take part in early preventive programs. The EI is one of the protective factors against Internet addiction, and it was not at a good level in some participants. There is a need for interventions to improve EI. Willingness to use m-learning (purposeful use of m-technology for educational purposes) is another protective factor against Internet addiction. Therefore, proper educational programs are needed to improve the media literacy and proper use of m-technology for educational purposes. Further studies are recommended to examine the effectiveness of improving EI and m-learning skills in lowering Internet addiction. 

On the other hand, our study has limitations. First, due to the nature of cross-sectional design, it is difficult to derive causal relationships from the analysis. A longitudinal study is recommended to determine the association and provide more evidence for possible causal interpretation. Second, since this research was conducted in a single university, the results may not be generalizable to other regions. To have accurate results, a larger group of students from other universities can participate in future studies. 

## Conclusion

A considerable number of the healthcare professional students had excessive and unnecessary use of the Internet. The EI is one of the protective factors against Internet addiction, and it was not at a good level in some participants. Willingness to use m-learning (purposeful use of m-technology for educational purposes) is another protective factor against Internet addiction. University students must be screened for high risk of Internet addiction, and early preventive programs must be taken. By taking proper measures to improve m-learning skills and EI, it would be possible to control Internet addiction to some extent. 

## Acknowledgement

We would like to acknowledge the authorities and staff of the study setting as well as all the students who participated in the study.

**Conflict of Interest:** None Declared.
